# Opportunity constraints and social inequalities in exercise participation: a lagged analysis using CFPS 2018–2022

**DOI:** 10.3389/fpubh.2026.1889919

**Published:** 2026-08-26

**Authors:** Xiwen Zhang, Wangjie Li

**Affiliations:** Nanyang Normal University, Nanyang, China

**Keywords:** CFPS, exercise participation, health behavior, longitudinal study, opportunity constraints, physical activity, social inequalities

## Abstract

**Background:**

Insufficient physical activity is socially patterned, but education, residence, work, health, and internet access are often modeled as separate covariates rather than as cumulative conditions that enable or restrict exercise.

**Methods:**

We analyzed individual-level longitudinal data from the China Family Panel Studies (CFPS) in 2018, 2020, and 2022. Two lagged person-period intervals were constructed: 2018–2020 and 2020–2022. The exposure was a standardized opportunity-constraint score ranging from 0 to 9, based on nine binary indicators across resource conditions, time conditions, health feasibility, and information access. The outcome was weekly exercise participation in the subsequent wave. Lagged logistic models adjusted for prior exercise participation, age, sex, marital status, period fixed effects, and province fixed effects; standard errors were clustered at the individual level.

**Results:**

The analytic sample included 32,306 person-period observations from 19,956 respondents. Subsequent weekly exercise participation was 43.94% in the low-constraint group, 27.65% in the moderate-constraint group, and 19.24% in the high-constraint group. In adjusted models, moderate and high constraints were associated with lower odds of subsequent participation than low constraints (OR = 0.555, 95% CI: 0.524–0.588; OR = 0.369, 95% CI: 0.340–0.401, respectively; both *p* < 0.001). The continuous-score model showed a similar inverse association (OR = 0.764, 95% CI: 0.750–0.779, *p* < 0.001). Resource-, time-, and information-access constraints were inversely associated with the outcome, whereas health-feasibility constraints were not independently associated after mutual adjustment. In an individual fixed-effects logit sensitivity model, the continuous score was not associated with subsequent participation (OR = 0.996, 95% CI: 0.927–1.070, *p* = 0.908).

**Conclusion:**

Multidimensional opportunity constraints were associated with a clear population-level gradient in subsequent weekly exercise participation. The null fixed-effects result indicates that this gradient should be interpreted as a between-person inequality rather than evidence of a short-term within-person causal association.

## Introduction

Insufficient physical activity remains a major modifiable risk factor for morbidity and premature mortality (1). The World Health Organization has positioned physical activity promotion as a central component of noncommunicable disease prevention and set a target to reduce insufficient physical activity by 15% by 2030 (2, 3). Its 2020 guidelines provide the basis for adult activity recommendations used in surveillance and intervention work (4, 5). Pooled estimates indicate that 31.3% of adults did not meet recommended activity levels in 2022 and that progress remains insufficient to meet the global target (6). Regular physical activity is associated with lower risks of all-cause mortality, cardiovascular disease, several cancers, depression, and functional decline (7–11). Understanding why exercise participation remains unequally distributed is therefore an important public-health and policy question.

In China, physical activity has declined alongside rapid economic development, urbanization, and lifestyle change (12). Activity is not distributed randomly across the population; international evidence documents systematic differences by national context, sex, age, and socioeconomic position (13–15), while Chinese studies identify residence, occupation, family socioeconomic conditions, and digital engagement as additional correlates of exercise disparities (16–18). Ecological models and reviews further suggest that participation reflects interacting individual, interpersonal, environmental, and policy conditions rather than motivation or health awareness alone (19, 20). Qualitative evidence among urban, educated postpartum women in Wuhan also illustrates how family responsibilities, gendered expectations, and limited support can obstruct the translation of exercise intentions into participation (21).

Despite this growing body of evidence, most existing quantitative studies suffer from two key limitations. First, they typically treat these diverse barriers as separate, independent covariates rather than as components of a cumulative system of constraints that jointly shape exercise opportunities. Second, most studies rely on cross-sectional data, which cannot establish the temporal order between constraints and exercise behavior. Therefore, building on health lifestyle theory, fundamental-cause theory, and cumulative-risk approaches—which collectively emphasize that health behavior arises from the dynamic interaction between individual agency and various structural factors (22–25)—we adopt an integrated “opportunity constraints” analytical framework. This framework conceptualizes exercise participation as a behavior that requires minimum practical preconditions, and our index measures whether respondents lack four critical sets of these preconditions: socioeconomic resources, discretionary time, health feasibility, and information access. Using nationally representative longitudinal data from the 2018, 2020, and 2022 waves of the China Family Panel Studies (CFPS), we employ a lagged person-period design to examine whether multidimensional opportunity constraints in one survey wave are associated with weekly exercise participation in subsequent waves. This study addresses three research questions: first, whether the continuous opportunity-constraint score is inversely associated with subsequent weekly exercise participation; second, whether groups with low, moderate, and high levels of constraints show a clear graded pattern of participation; and third, whether this gradient exists across sex, urban–rural residence, and age subgroups.

## Materials and methods

### Data source

We used individual-level longitudinal data from the 2018, 2020, and 2022 waves of the China Family Panel Studies (CFPS). The CFPS is a nationally representative biennial longitudinal survey administered by the Institute of Social Science Survey at Peking University. Its baseline sample covered 25 provinces, municipalities, and autonomous regions and used a multistage probability design with implicit stratification and probability-proportional-to-size sampling (26, 27). The survey collects socioeconomic, demographic, family, and health information, although some questionnaire items differ across waves. The original CFPS protocol was approved by the Peking University Biomedical Institutional Review Board (IRB00001052-14010), and participants provided written informed consent (28). De-identified public-use data are available through the official CFPS data platform subject to registration and data-use requirements.

### Sample selection and data processing

**Figure 1:**
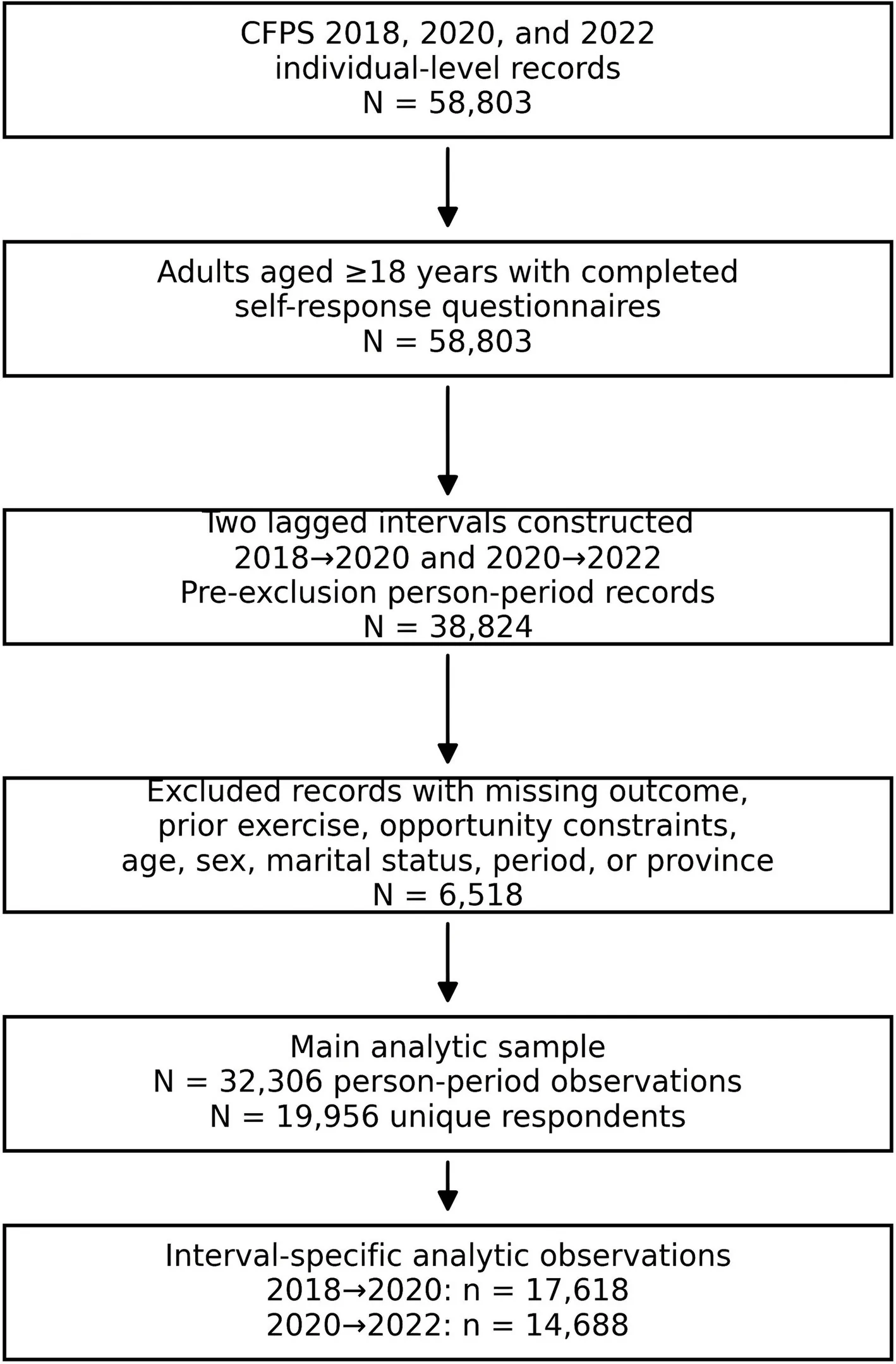
Sample selection flowchart. The final analytic sample included 32,306 person-period observations from 19,956 respondents across the 2018–2020 and 2020–2022 lagged intervals.

Sample processing proceeded in four steps. First, we loaded the 2018, 2020, and 2022 individual-level files and retained adults aged 18 years or older who completed the self-response questionnaire. Second, we constructed weekly exercise participation, covariates, and nine opportunity-constraint indicators using year-specific questionnaire items and Stata labels. Third, we linked adjacent waves using the unique personal identifier and generated two lagged intervals: 2018–2020 and 2020–2022. Fourth, we excluded observations missing the outcome, prior exercise participation, opportunity-constraint score or group, age, sex, marital status, period, or province. Figure 1 summarizes the analytic sample selection.

### Measures

The outcome was weekly exercise participation in the subsequent survey wave. In 2018, item QP701 recorded the number of exercise sessions during the previous week, and respondents reporting at least one session were classified as participating weekly. In 2020 and 2022, item QP701N assessed the usual frequency of sports, fitness, and leisure activities during the previous 12 months; response categories corresponding to participation at least once per week were coded as 1, and lower frequencies or no participation were coded as 0. Negative response codes, including “do not know,” “refused,” and “not applicable,” were treated as missing. The threshold of at least once per week was selected because it could be identified in all three waves. This harmonization aligned the minimum frequency threshold, although differences in item wording and recall period remained.

The opportunity-constraint index was developed to capture cumulative practical conditions that may restrict the translation of an intention to exercise into weekly participation. Drawing on health lifestyle theory, fundamental-cause theory, and cumulative-risk approaches, we conceptualized exercise participation as requiring several distinct but complementary preconditions (22–25). Resource conditions reflect access to socioeconomic and social resources that may facilitate the use of exercise spaces, equipment, and supportive networks. Time conditions represent the availability of discretionary time outside employment and extended working hours. Health feasibility reflects whether an individual’s physical condition permits regular participation, whereas information access represents the ability to obtain exercise-related knowledge, activity information, and digital health resources. These domains represent practical pathways through which social conditions may enable or restrict exercise participation (19, 29–35).

The index follows a cumulative-indicator approach to summarize opportunity constraints across domains. The nine indicators represent distinct practical constraints that may operate independently or accumulate within the same individual. Each indicator was coded as 1 when the constraint was present and 0 otherwise and was assigned equal weight. Equal weighting was chosen because no externally validated theoretical or empirical basis was available for assigning a fixed greater weight to any one constraint; it also preserves transparency, interpretability, and reproducibility across survey waves (36, 37). We additionally constructed a principal-component-weighted index to assess whether the findings depended on the weighting decision.

Resource-condition constraints included low education, rural residence, and low subjective social status. Time-condition constraints included current employment and long working hours. Health-feasibility constraints included poor self-rated health, chronic disease, and recent hospitalization. Information-access constraints were defined as neither using mobile internet nor computer internet. For each person-period observation, we calculated the number of constraints present, divided it by the number of valid indicators, and multiplied by nine to retain a common 0–9 scale:


$$\text{constraint}\_{\text{score}}_{\mathrm{it}}=\frac{\text{constraint}\_{\mathrm{raw}}_{\mathrm{it}}}{\text{constraint}\_{\text{valid}}_{\mathrm{it}}}\times 9.$$

Full coding information and indicator distributions are provided in Supplementary Table S1.

**Figure 2:**
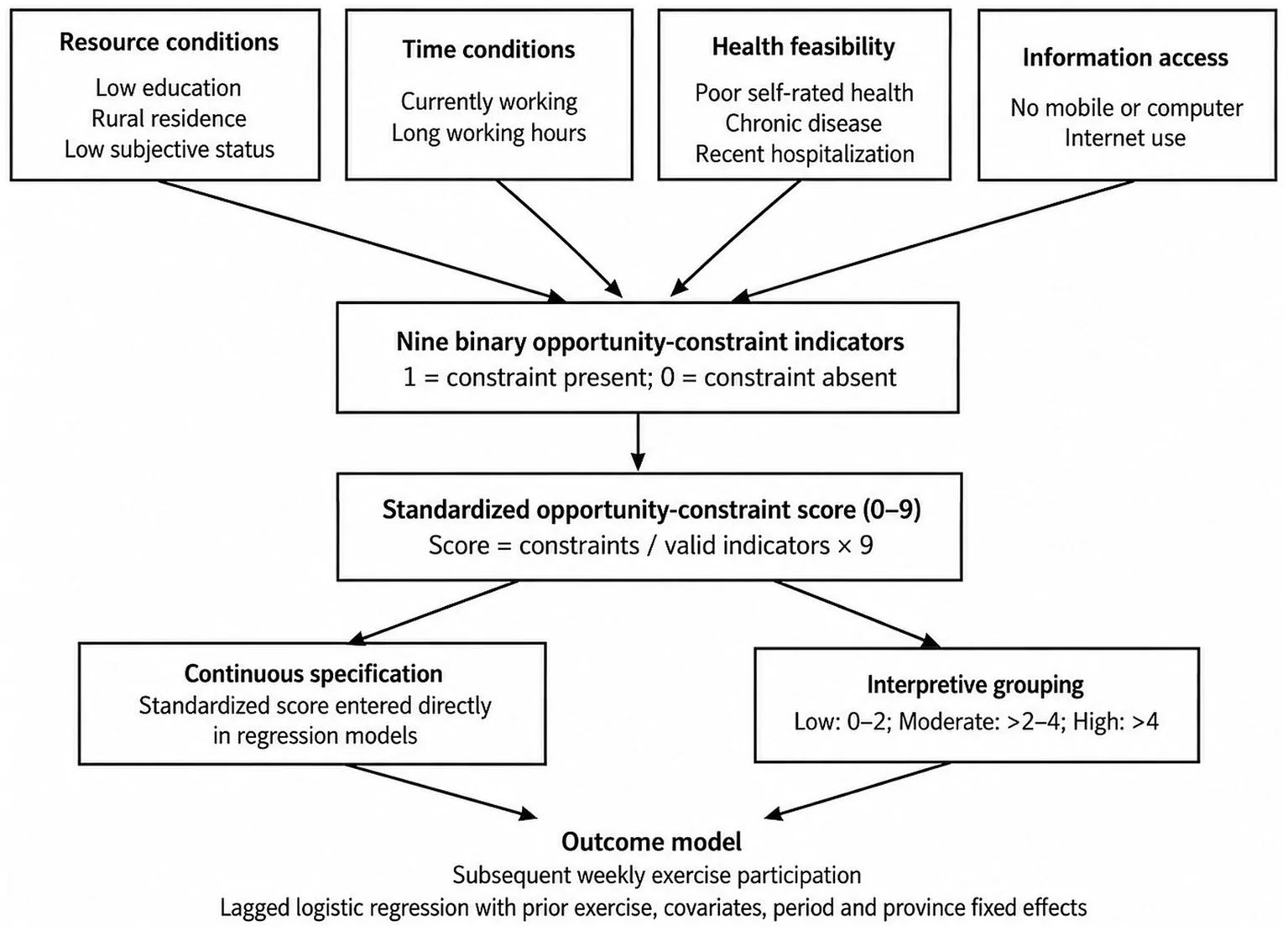
Construction of the multidimensional opportunity-constraint score and analytic framework. The index combines nine binary indicators across resource conditions, time conditions, health feasibility, and information access. Indicators were equally weighted to preserve transparency, interpretability, and reproducibility because no externally validated basis was available for assigning fixed differential weights. Robustness to alternative weighting was assessed using a principal-component-weighted specification. The standardized score was modeled continuously and grouped into low, moderate, and high levels for interpretive presentation.

The score was computed only when at least seven of the nine indicators were valid. For interpretive presentation, scores of 0–2 were classified as low constraints, >2–4 as moderate constraints, and >4 as high constraints. These categories distinguish relatively limited, intermediate, and higher cumulative burden; they are descriptive groupings rather than externally validated thresholds. The continuous score was therefore the primary specification. Because the category boundaries were not externally validated, we repeated the grouped model after shifting both cut points downward by 0.5 points and upward by 0.5 points. Figure 2 shows the index construction and analytic framework.

### Statistical analysis

We used a lagged person-period design in which opportunity constraints in wave t were modeled as the exposure and weekly exercise participation in wave t + 1 as the outcome. The main specification used the continuous standardized score, and the grouped specification was used to display interpretable gradients. Lagged logistic regression models adjusted for prior weekly exercise participation, age, sex, marital status, period fixed effects, and province fixed effects. Standard errors were clustered at the individual level because respondents could contribute up to two observations (38).

We specified the following lagged logistic regression model to estimate the association between prior opportunity constraints and subsequent weekly exercise participation:


$$\begin{array}{l} \text{logit}\{\mathrm{Pr}(Y_{i,t+1}=1)\}=\alpha +\beta {\text{Constraint}}_{i,t}\\ +\theta {\text{Exercise}}_{i,t}+\gamma^{\prime }X_{i,t}+\delta_{t}+\mu_{p}+\varepsilon_{i,t} \end{array}$$

where *Yᵢ*,*ₜ*₊₁ denotes weekly exercise participation for individual i in the subsequent survey wave; Constraint*ᵢ*,*ₜ* denotes the opportunity-constraint measure in wave t, entered as the continuous standardized score or categorical constraint groups; Exercise*ᵢ*,*ₜ* denotes weekly exercise participation in wave t; X*ᵢ*,*ₜ* includes age, sex, and marital status; *δₜ* denotes period fixed effects; *μₚ* denotes province fixed effects; and *εᵢ*,*ₜ* is the error term. We report odds ratios (ORs) with 95% confidence intervals (CIs), adjusted predicted probabilities, and average marginal effects where appropriate (39, 40). As supplementary in-sample assessments, we calculated the area under the receiver operating characteristic curve (AUC) and Brier score for each main model. For the primary continuous-score model, calibration was examined by comparing observed and predicted probabilities across deciles of predicted probability (41, 42). These metrics describe in-sample performance and do not constitute external validation. Analyses were performed using Stata/MP 19.5 (StataCorp, College Station, TX, United States).

Sensitivity analyses assessed the influence of index composition, the complete nine-indicator requirement, observation interval, principal-component weighting, alternative category cut points, and individual fixed effects. The alternative-cut-point models shifted both original boundaries downward by 0.5 points (1.5 and 3.5) and upward by 0.5 points (2.5 and 4.5) while retaining the same sample and model specification. The fixed-effects logit model was interpreted as a distinct estimand: it assesses whether within-person changes in constraints are associated with within-person changes in subsequent exercise participation, whereas the primary models estimate population-level between-person differences. Subgroup analyses were conducted by sex, urban–rural residence, and age. For the urban–rural subgroup analysis, rural residence was removed from the index before reconstructing the constraint groups.

## Results

### Descriptive characteristics

The final analytic sample included 32,306 person-period observations from 19,956 respondents. Of these, 12,675 observations were classified as low constraints, 13,354 as moderate constraints, and 6,277 as high constraints. The mean standardized opportunity-constraint score was 1.38 in the low-constraint group, 3.37 in the moderate-constraint group, and 5.51 in the high-constraint group. Domain-specific constraint scores also increased across the three groups, indicating that the grouping captured a rising cumulative burden (Table 1). Full coding and distributional information for the nine indicators is reported in Supplementary Table S1.

**Table 1:** Selected characteristics of the analytic sample by opportunity-constraint group.

Variable	Overall	Low constraints	Moderate constraints	High constraints	*p* value	Valid *N*
Age, years	45.74 (15.48)	40.12 (15.15)	47.04 (15.10)	54.35 (12.01)	<0.001	32,306
Standardized opportunity-constraint score	3.01 (1.66)	1.38 (0.68)	3.37 (0.54)	5.51 (0.81)	<0.001	32,306
Resource-condition constraint score	1.06 (0.86)	0.42 (0.56)	1.23 (0.69)	1.97 (0.67)	<0.001	32,296
Time-condition constraint score	1.10 (0.79)	0.75 (0.71)	1.25 (0.79)	1.42 (0.72)	<0.001	30,961
Health-feasibility constraint score	0.52 (0.80)	0.14 (0.39)	0.49 (0.72)	1.35 (0.98)	<0.001	32,306
Information-access constraint score	0.39 (0.49)	0.12 (0.32)	0.44 (0.50)	0.81 (0.39)	<0.001	32,305
Subsequent weekly exercise participation	10,470 (32.4%)	5,569 (43.9%)	3,693 (27.7%)	1,208 (19.2%)	<0.001	32,306
Prior weekly exercise participation	13,463 (41.7%)	6,570 (51.8%)	4,975 (37.3%)	1,918 (30.6%)	<0.001	32,306
Male	16,083 (49.8%)	5,842 (46.1%)	7,205 (54.0%)	3,036 (48.4%)	<0.001	32,306
Married/cohabiting	26,204 (81.1%)	9,610 (75.8%)	11,048 (82.7%)	5,546 (88.4%)	<0.001	32,306
2018–2020	17,618 (54.5%)	6,491 (51.2%)	7,242 (54.2%)	3,885 (61.9%)	<0.001	32,306
2020–2022	14,688 (45.5%)	6,184 (48.8%)	6,112 (45.8%)	2,392 (38.1%)		32,306

The unit of analysis is the person-period observation. Continuous variables are reported as mean (SD); binary variables and observation intervals are reported as *n* (%). Full descriptive information for all opportunity-constraint indicators is reported in Supplementary Table S1.

Overall, 10,470 observations reported weekly exercise participation in the subsequent wave, corresponding to 32.41% of the analytic sample. Participation declined from 43.94% in the low-constraint group to 27.65% in the moderate-constraint group and 19.24% in the high-constraint group. These descriptive differences were unadjusted but showed the expected descending gradient.

### Lagged logistic regression models

Table 2 presents the adjusted lagged logistic models. Relative to the low-constraint group, the moderate-constraint group had lower odds of subsequent weekly exercise participation (OR = 0.555, 95% CI: 0.524–0.588, *p* < 0.001), and the high-constraint group had even lower odds (OR = 0.369, 95% CI: 0.340–0.401, *p* < 0.001). Prior weekly exercise participation was strongly associated with subsequent participation (OR = 4.159, 95% CI: 3.930–4.401, *p* < 0.001).

**Table 2:** Lagged logistic regression models for opportunity constraints and subsequent weekly exercise participation.

Variable	Model 1: constraint groups	Model 2: continuous score	Model 3: domain-specific model
Moderate constraints (reference: low constraints)	OR = 0.555 95% CI: 0.524–0.588 *p* < 0.001	—	—
High constraints (reference: low constraints)	OR = 0.369 95% CI: 0.340–0.401 *p* < 0.001	—	—
Standardized opportunity-constraint score	—	OR = 0.764 95% CI: 0.750–0.779 p < 0.001	—
Resource-condition constraints	—	—	OR = 0.664 95% CI: 0.641–0.688 *p* < 0.001
Time-condition constraints	—	—	OR = 0.807 95% CI: 0.778–0.837 *p* < 0.001
Health-feasibility constraints	—	—	OR = 0.993 95% CI: 0.959–1.029 *p* = 0.712
Information-access constraints	—	—	OR = 0.600 95% CI: 0.560–0.644 *p* < 0.001
Prior weekly exercise participation	OR = 4.159 95% CI: 3.930–4.401 *p* < 0.001	Controlled	Controlled
Observations	32,306	32,306	30,950
Individual clusters	19,956	19,956	19,095
Pseudo *R*^2^	0.1287	0.1329	0.1377
AUC	0.7387	0.7423	0.7460
Brier score	0.1840	0.1829	0.1779

The outcome is weekly exercise participation in the subsequent wave. All models adjusted for prior exercise participation, age, sex, marital status, period fixed effects, and province fixed effects. Standard errors were clustered at the individual level. AUC denotes the area under the receiver operating characteristic curve. The Brier score is the mean squared difference between observed outcomes and predicted probabilities; lower values indicate smaller in-sample probability error. These performance measures are descriptive and do not constitute external validation. OR, odds ratio; CI, confidence interval.

When the continuous score was entered directly, each one-point increase in the standardized opportunity-constraint score was associated with lower odds of subsequent weekly exercise participation (OR = 0.764, 95% CI: 0.750–0.779, *p* < 0.001). Adjusted predicted probabilities declined from 48.38% at a score of 0 to 31.66% at 3, 18.37% at 6, and 9.61% at 9. Complete predicted probabilities across the 0–9 score range are reported in Supplementary Table S2.

In the domain-specific model, resource-condition constraints (OR = 0.664, 95% CI: 0.641–0.688, *p* < 0.001), time-condition constraints (OR = 0.807, 95% CI: 0.778–0.837, *p* < 0.001), and information-access constraints (OR = 0.600, 95% CI: 0.560–0.644, *p* < 0.001) were inversely associated with subsequent weekly exercise participation. Health-feasibility constraints were not independently associated after mutual adjustment (OR = 0.993, 95% CI: 0.959–1.029, *p* = 0.712).

The AUC values for Models 1–3 were 0.739, 0.742, and 0.746, and the corresponding Brier scores were 0.184, 0.183, and 0.178 (Table 2). Because Model 3 used a slightly smaller analytic sample, these values are reported as descriptive in-sample measures rather than formal comparisons between models. Calibration of the continuous-score model was generally close to the ideal line across deciles of predicted probability (Supplementary Figure S1).

### Sensitivity and subgroup analyses

Period-specific analyses retained the same descending ordering in both observation intervals, although absolute probabilities differed. In 2018–2020, adjusted predicted probabilities were 38.22, 25.34, and 18.35% in the low-, moderate-, and high-constraint groups; in 2020–2022, the corresponding probabilities were 43.64, 33.75, and 27.85% (Supplementary Table S2). The principal-component-weighted index produced a substantively consistent inverse association (OR = 0.803, 95% CI: 0.775–0.832, *p* < 0.001; Supplementary Table S3). The gradient was also retained after shifting both category cut points downward by 0.5 points (moderate vs. low: OR = 0.591, 95% CI: 0.553–0.631; high vs. low: OR = 0.324, 95% CI: 0.300–0.350) and upward by 0.5 points (moderate vs. low: OR = 0.555, 95% CI: 0.524–0.588; high vs. low: OR = 0.369, 95% CI: 0.339–0.401; all *p* < 0.001; Supplementary Table S4).

The individual fixed-effects logit model produced a null result. The standardized opportunity-constraint score was not associated with subsequent weekly exercise participation (OR = 0.996, 95% CI: 0.927–1.070, *p* = 0.908). This model retained 6,634 person-period observations from 3,317 individuals because respondents without within-person outcome variation were excluded. The null finding places an important boundary on the primary estimates: the observed gradient should be interpreted mainly as a population-level difference between people facing different levels of constraints and not as evidence that short-term increases in constraints within the same individual cause subsequent reductions in exercise participation.

**Figure 3:**
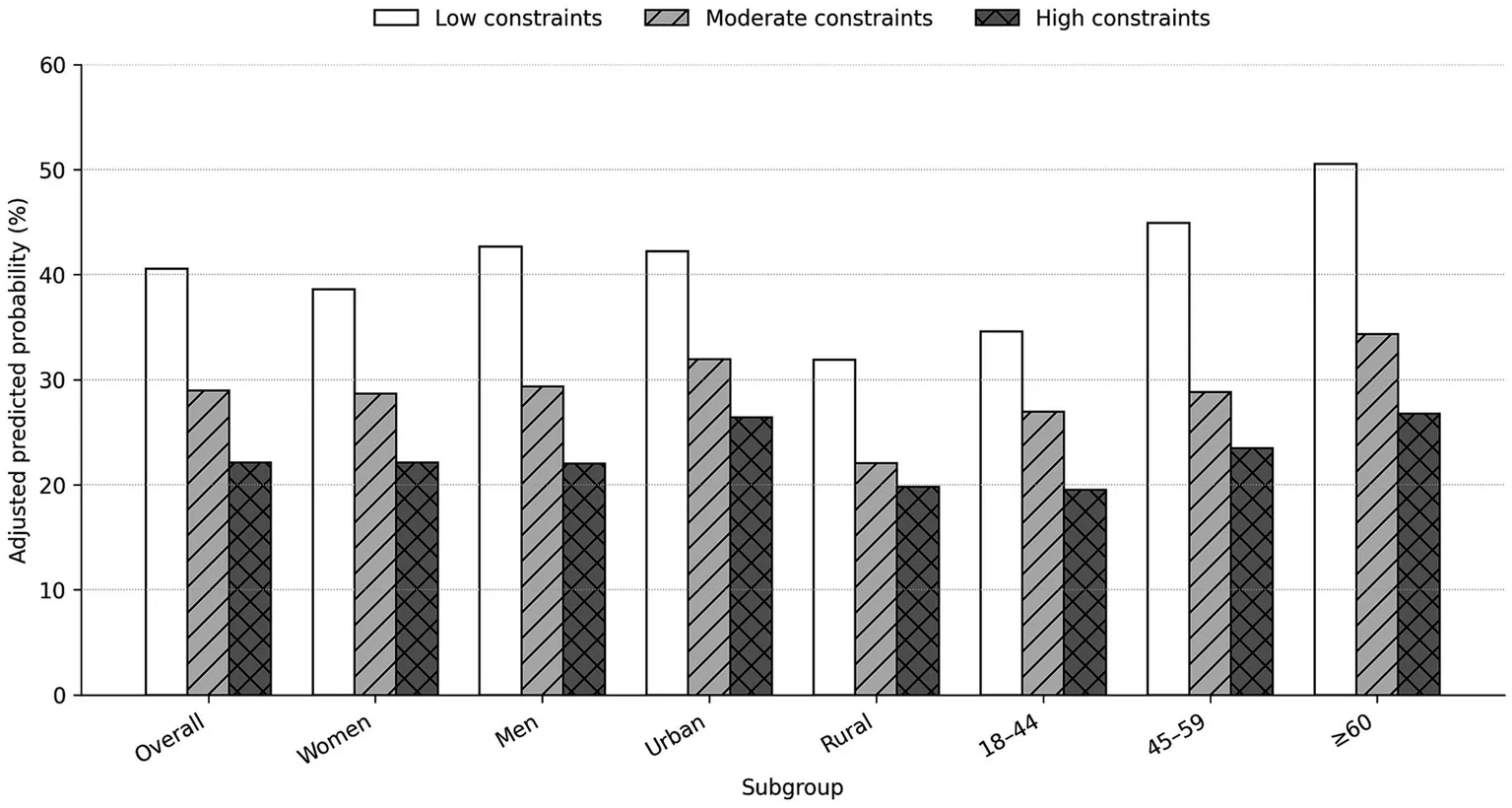
Adjusted predicted probabilities of subsequent weekly exercise participation by opportunity-constraint group and subgroup. The urban–rural subgroup analysis reconstructed the constraint groups after excluding rural residence from the index.

Figure 3 shows adjusted predicted probabilities across subgroups. The descending gradient was observed among women and men, urban and rural residents, and adults aged 18–44, 45–59, and 60 years or older. Rural residents had lower predicted probabilities overall than urban residents after reconstructing the index without the rural-residence item. Numeric subgroup estimates are reported in Supplementary Table S2.

## Discussion

This study identified a consistent population-level gradient between opportunity constraints and subsequent weekly exercise participation among Chinese adults. Respondents with greater cumulative constraints were less likely to participate in weekly exercise across the grouped, continuous-score, domain-specific, and subgroup analyses. Resource, time, and information-access constraints contributed most clearly to this pattern, whereas health-feasibility constraints were not independently associated after mutual adjustment. These findings indicate that weekly exercise participation is socially patterned by the practical conditions under which individuals attempt to maintain regular activity.

The individual fixed-effects model qualifies the primary findings. After controlling for time-invariant individual characteristics, within-person changes in the opportunity-constraint score were not associated with subsequent changes in weekly exercise participation. The primary gradient should therefore be interpreted mainly as a difference between people facing different levels of cumulative constraints. It does not establish that a short-term increase in constraints within the same individual causes a subsequent reduction in participation. Several index components, including education and urban–rural residence, changed little across adjacent waves, and the fixed-effects model retained only respondents with outcome variation. These features may limit within-person information, but they do not justify dismissing the null result.

The findings are consistent with theoretical approaches that locate health behavior at the intersection of individual agency and social structure (22, 23). Health lifestyle theory emphasizes that behavioral choices develop within socially patterned living conditions, while fundamental-cause theory explains how flexible socioeconomic resources can support health-protective practices. A cumulative-risk perspective further suggests that disadvantages frequently cluster rather than occur in isolation (24, 25). Applied to exercise participation, these perspectives indicate that limited resources, restricted discretionary time, and unequal access to information may jointly narrow the range of feasible behavioral choices.

The domain-specific results suggest several plausible pathways. Resource constraints may limit access to safe and affordable exercise spaces, equipment, organized activities, and supportive social networks (19, 29, 30). Time constraints may operate through limited discretionary time among people working long or irregular hours. Occupational physical activity may not compensate for this limitation because it differs from voluntary leisure-time exercise in intensity, recovery, and individual control (31–33). Information-access constraints may reduce exposure to exercise knowledge, community activity information, online classes, and digital health services among people with limited internet access or digital skills (18, 34, 35).

Exercise opportunities may also depend on contextual conditions that were not measured consistently across CFPS waves. Neighborhood walkability, the proximity and affordability of recreational facilities, perceived environmental safety, and community organization may determine whether available resources can be converted into participation (19, 20, 29, 30). Caregiving demands may reduce discretionary time even among people not classified as working long hours, while cultural expectations concerning family roles and gender may influence whether exercise is regarded as a legitimate use of time (21). These conditions may interact with the measured resource, time, health, and information constraints rather than operate independently.

This study has several limitations. First, exercise participation was assessed using items with different wording and reference periods. The 2018 item recorded sessions during the previous week, whereas the 2020 and 2022 items assessed usual participation in sports, fitness, and leisure activities during the previous 12 months. Harmonizing the measures to a threshold of at least once per week improved comparability but did not establish complete measurement equivalence. The 2018 measure may be more sensitive to short-term fluctuations, while the later measures may be affected by longer-term recall and the averaging of irregular activity. Respondents close to the threshold could therefore be classified differently across waves, and the direction of any resulting bias cannot be determined. The period-specific analyses retained the same descending gradient in both intervals, reducing concern that the overall pattern was confined to one questionnaire version but not eliminating this limitation. Second, the index did not capture all contextual and household conditions relevant to participation, including neighborhood walkability, facility proximity, quality and affordability, perceived safety, caregiving demands, community programs, cultural attitudes, or local policy implementation. Some omitted conditions may confound the observed associations, while others may modify how measured constraints translate into behavior. Residual confounding and incomplete representation of the opportunity environment therefore remain possible, and the direction and magnitude of any resulting bias cannot be determined. Third, the low-, moderate-, and high-constraint cut points were selected for interpretive presentation and have not been externally validated. The continuous-score model was the primary specification, and shifted-cut-point analyses retained the gradient, but the categories should not be interpreted as diagnostic thresholds. Fourth, the observational lagged design cannot establish causality. The null fixed-effects estimate further precludes interpreting the primary association as a short-term within-person causal effect. Future studies could use target trial emulation, natural experiments, or intervention designs to assess the effects of reducing specific constraints (43–46).

These findings suggest that reducing inequalities in weekly exercise participation requires attention to the structural conditions that make exercise feasible. At the population level, resource constraints could be addressed by expanding accessible, free or low-cost exercise facilities in rural areas and under-resourced urban communities, improving walking and cycling environments, and increasing public access to sports venues and school facilities (29, 30). Time constraints are unlikely to be resolved through health education alone. Oversight of excessive working hours, predictable rest time, and workplace arrangements that provide usable opportunities for exercise may be required. Information constraints could be addressed through improved digital access combined with offline exercise information, community outreach, and in-person guidance for people with limited internet access or digital skills (18, 34, 35).

At the individual level, community exercise programs, digital-skills support, guidance from social sports instructors, and adapted exercise programs may help older adults, people with chronic conditions, and individuals with limited exercise experience use available opportunities. Such support may facilitate participation but cannot replace measures addressing unequal access to time, facilities, affordable services, and information. Future research should evaluate whether specific structural and individual-level interventions reduce participation inequalities and should examine how constraints accumulate across the life course.

## Conclusion

Using CFPS 2018–2022 data and a lagged person-period design, this study found that greater multidimensional opportunity constraints were associated with lower subsequent weekly exercise participation. The association was observed in grouped, continuous-score, domain-specific, and subgroup analyses, and it remained under alternative weighting and category cut points.

The results indicate a population-level between-person inequality in exercise participation across levels of cumulative opportunity constraints. The null individual fixed-effects estimate means that the observed association should not be interpreted as evidence that short-term changes in constraints cause corresponding changes in participation. Public-health strategies may therefore consider both individual support and the unequal resource, time, and information conditions that shape opportunities to exercise. Causal effects of reducing specific constraints require evaluation in stronger quasi-experimental or intervention designs.

## Data Availability

The data analyzed in this study is subject to the following licenses/restrictions: The datasets analyzed in this study were obtained from the China Family Panel Studies conducted by the Institute of Social Science Survey, Peking University. Researchers may apply for access through the official CFPS data platform in accordance with the provider’s registration and use policies. Requests to access these datasets should be directed to https://cfpsdata.pku.edu.cn/#/home.
