# Supplementary material for: Opportunity constraints and social inequalities in exercise participation: a lagged analysis using CFPS 2018–2022
Source: Front Public Health. 2026 Aug 26;14:1889919. doi: 10.3389/fpubh.2026.1889919 (PMC13553914; doi:10.3389/fpubh.2026.1889919)
Supplement: Supplementary file 1 [file Data_Sheet_1.docx]

# Supplementary material

Supplementary Table S1. Variable coding and full distribution of the nine opportunity-constraint indicators.

| **Domain** | **Indicator** | **Coding** | **Overall** | **Low constraints** | **Moderate constraints** | **High constraints** | **p value** | **Valid N** |
| --- | --- | --- | --- | --- | --- | --- | --- | --- |
| Resource conditions | Low education | 1 = <9 years of schooling; 0 = ≥9 years | 10,876 (34.0%) | 928 (7.3%) | 5,018 (38.0%) | 4,930 (80.2%) | <0.001 | 32,007 |
| Resource conditions | Rural residence | 1 = rural residence; 0 = urban residence | 15,164 (48.1%) | 2,646 (21.3%) | 7,543 (58.4%) | 4,975 (80.7%) | <0.001 | 31,500 |
| Resource conditions | Low subjective social status | 1 = QN8012 category 1 or 2; 0 = categories 3–5 | 7,728 (24.0%) | 1,773 (14.0%) | 3,683 (27.6%) | 2,272 (36.4%) | <0.001 | 32,242 |
| Time conditions | Currently employed | 1 = currently working (QGB1 = 1); 0 = not currently working | 22,536 (72.8%) | 6,894 (59.3%) | 10,240 (78.4%) | 5,402 (86.1%) | <0.001 | 30,961 |
| Time conditions | Long working hours | 1 = ≥50 h/week among workers; 0 = <50 h/week or not working | 10,359 (34.6%) | 1,691 (14.7%) | 5,598 (44.4%) | 3,070 (52.5%) | <0.001 | 29,934 |
| Health feasibility | Poor self-rated health | 1 = QP201 category 4 or 5; 0 = categories 1–3 | 8,310 (25.7%) | 906 (7.1%) | 3,383 (25.3%) | 4,021 (64.1%) | <0.001 | 32,305 |
| Health feasibility | Chronic disease | 1 = chronic disease present (QP401 = 1); 0 = absent | 5,050 (15.6%) | 563 (4.4%) | 1,941 (14.5%) | 2,546 (40.6%) | <0.001 | 32,303 |
| Health feasibility | Recent hospitalization | 1 = recent hospitalization (QC401 = 1); 0 = none | 3,485 (10.8%) | 345 (2.7%) | 1,259 (9.4%) | 1,881 (30.0%) | <0.001 | 32,305 |
| Information access | No mobile or computer internet use | 1 = both mobile and computer internet use reported as no; 0 = either mode reported as yes; indeterminate responses retained as missing | 12,470 (38.6%) | 1,487 (11.7%) | 5,892 (44.1%) | 5,091 (81.1%) | <0.001 | 32,305 |

Note: The unit of analysis is the person-period observation. Percentages are calculated within the relevant column. The standardized opportunity-constraint score was calculated as (number of constraints present / number of valid indicators) × 9 and was computed only when at least seven of the nine indicators were valid.

Supplementary Table S2. Additional adjusted predicted probabilities from lagged models.

Panel A. Predicted probabilities across the standardized opportunity-constraint score.

| Standardized score | Adjusted predicted probability | 95% CI |
| --- | --- | --- |
| 0 | 48.38% | 47.12%–49.63% |
| 1 | 42.58% | 41.69%–43.47% |
| 2 | 36.97% | 36.37%–37.56% |
| 3 | 31.66% | 31.17%–32.15% |
| 4 | 26.75% | 26.15%–27.34% |
| 5 | 22.30% | 21.54%–23.06% |
| 6 | 18.37% | 17.47%–19.26% |
| 7 | 14.95% | 13.97%–15.92% |
| 8 | 12.04% | 11.04%–13.04% |
| 9 | 9.61% | 8.63%–10.58% |

Note for Panel A: Predicted probabilities were estimated from the continuous standardized opportunity-constraint score model adjusted for prior exercise participation, age, sex, marital status, period fixed effects, and province fixed effects.

Panel B. Period-specific adjusted predicted probabilities by opportunity-constraint group.

| Lagged interval | Low constraints | Moderate constraints | High constraints |
| --- | --- | --- | --- |
| 2018–2020 | 38.22% | 25.34% | 18.35% |
| 2020–2022 | 43.64% | 33.75% | 27.85% |

Note for Panel B: Estimates summarize the opportunity-constraint gradient separately for the 2018–2020 and 2020–2022 lagged intervals.

Panel C. Numeric subgroup estimates corresponding to main Figure 3.

| Stratification variable | Subgroup | Low constraints | Moderate constraints | High constraints |
| --- | --- | --- | --- | --- |
| Overall | All respondents | 40.56% | 28.95% | 22.09% |
| Sex | Women | 38.58% | 28.67% | 22.12% |
| Sex | Men | 42.66% | 29.32% | 22.00% |
| Residence | Urban | 42.21% | 31.94% | 26.39% |
| Residence | Rural | 31.89% | 22.04% | 19.79% |
| Age | 18–44 years | 34.58% | 26.94% | 19.51% |
| Age | 45–59 years | 44.91% | 28.81% | 23.48% |
| Age | ≥60 years | 50.52% | 34.33% | 26.77% |

Note for Panel C: The total, sex, and age subgroup estimates used the standardized opportunity-constraint groups. The urban-rural subgroup analysis used groups reconstructed after excluding rural residence from the index. Values are adjusted predicted probabilities.

Supplementary Table S3. Sensitivity analyses of the opportunity-constraint index.

| **Sensitivity model** | **Core variable** | **OR** | **95% CI** | **p value** | **N** | **Individuals/groups** |
| --- | --- | --- | --- | --- | --- | --- |
| Main continuous-score model | Standardized opportunity-constraint score (0–9) | 0.764 | 0.750–0.779 | <0.001 | 32,306 | 19,956 |
| Excluding the health domain | Non-health opportunity-constraint score (0–6) | 0.703 | 0.688–0.719 | <0.001 | 30,930 | 19,084 |
| Complete nine-indicator sample | Standardized opportunity-constraint score | 0.768 | 0.753–0.783 | <0.001 | 28,790 | 18,283 |
| Excluding the resource domain | Opportunity-constraint score | 0.810 | 0.790–0.830 | <0.001 | 30,961 | 19,104 |
| Excluding the time domain | Opportunity-constraint score | 0.776 | 0.760–0.793 | <0.001 | 32,296 | 19,948 |
| Excluding the information domain | Opportunity-constraint score | 0.772 | 0.757–0.788 | <0.001 | 32,306 | 19,956 |
| PCA-weighted index | Standardized first principal-component score | 0.803 | 0.775–0.832 | <0.001 | 28,790 | 18,283 |
| Individual fixed-effects logit | Standardized opportunity-constraint score | 0.996 | 0.927–1.070 | 0.908 | 6,634 | 3,317 |

Note: Except for the individual fixed-effects logit model, all models adjusted for prior exercise participation, age, sex, marital status, period fixed effects, and province fixed effects, with standard errors clustered at the individual level. The fixed-effects logit model used within-person outcome variation and excluded respondents without within-person outcome changes.

Supplementary Table S4. Sensitivity analysis using alternative opportunity-constraint category cut points.

| Grouping rule | Moderate vs low, OR (95% CI) | High vs low, OR (95% CI) | Observations | Individual clusters |
| --- | --- | --- | --- | --- |
| Original cut points: 2.0 and 4.0 | 0.555 (0.524–0.588) | 0.369 (0.340–0.401) | 32,306 | 19,956 |
| Both cut points shifted down by 0.5: 1.5 and 3.5 | 0.591 (0.553–0.631) | 0.324 (0.300–0.350) | 32,306 | 19,956 |
| Both cut points shifted up by 0.5: 2.5 and 4.5 | 0.555 (0.524–0.588) | 0.369 (0.339–0.401) | 32,306 | 19,956 |

Note: The fully adjusted lagged logistic model, analytic sample, fixed effects, and individual-level clustered standard errors were held constant while both category boundaries were shifted. All reported comparisons had p < 0.001. OR, odds ratio; CI, confidence interval.

Supplementary Figure S1. In-sample calibration of the continuous opportunity-constraint score model.

Note: Points compare the observed proportion with the mean predicted probability within deciles of predicted probability. The dashed diagonal indicates ideal calibration. Predicted probabilities came from the lagged logistic model including the continuous standardized opportunity-constraint score and adjusted for prior weekly exercise participation, age, sex, marital status, period fixed effects, and province fixed effects.
